# The Efficacy of Parent Training Interventions for Disruptive Behavior Disorders in Treating Untargeted Comorbid Internalizing Symptoms in Children and Adolescents: A Systematic Review

**DOI:** 10.1007/s10567-021-00349-1

**Published:** 2021-05-15

**Authors:** Eleni Zarakoviti, Roz Shafran, Danai Papadimitriou, Sophie D. Bennett

**Affiliations:** 1 Population, Policy and Practice Research and Teaching Department, UCL Great Ormond Street Institute of Child Health, 30 Guilford Street, London, WC1N 1EH UK; 2 Great Ormond Street Hospital NHS Foundation Trust, Great Ormond Street, London, WC1N 3JH UK; 3 Imperial College London, South Kensington, London, SW7 2BU UK

**Keywords:** Disruptive behavior disorder, Intervention, Parent training, Children, Adolescents

## Abstract

Disruptive behavior disorders (DBDs) are among the primary reasons for child and youth referrals to mental health services and are linked to poor adult outcomes including antisocial behavior disorder. Research indicates a high incidence of internalizing problems in those with DBDs and those who have DBDs with cooccurring internalizing problems may have more severe later outcomes. Interventions targeted at internalizing symptoms have been found to also reduce comorbid externalizing problems. The impact of treatments for DBDs on comorbid internalizing disorders is not known. Databases PsycINFO, EMBASE and MEDLINE were systematically searched based on the Cochrane guidelines for systematic reviews. Records were independently reviewed by two reviewers. 12 papers were deemed eligible. A quality assessment of the selected studies was conducted independently by both reviewers. The 12 studies included 1334 young people with a mean age of 5 years. The parent training interventions assessed were the Incredible Years (6/12 studies), Triple-P (5/12) and Tuning In To Kids (1/12). 11 of the 12 studies reported significant reductions in primary externalizing behavior problems and DBDs. 7 studies reported significant reductions in internalizing symptoms. Mechanisms of change, clinical implications and directions for future research are discussed.

## Introduction

Disruptive Behavior Disorders (DBDs) are among the most frequent reasons for child and adolescent referrals to mental health services (Hood & Eyberg, 2003; Katzmann et al., 2019). DBDs often develop in childhood or adolescence (Turner, Hu, Villa and Nock 2018) and have been linked to high rates of criminality and antisocial personality disorder in adulthood, as well as to poor outcomes in terms of employment and social relations (Gacono, Nieberding, Owen, Rubel, & Bodholdt, 2001; Bjorseth & Wichstrom, 2016).

Research indicates a high incidence of internalizing problems in those with DBDs, estimated at around 20% (Polier et al. 2012; Stalk et al. 2015) and those who have DBDs with co-occurring internalizing problems may have more severe later outcomes (Eisenberg et al. 2001; Fraire & Ollendick, 2013). Conversely, it is also possible for anxiety disorders to be a protective factor for young people with DBDs (Cunningham & Ollendick, 2010) and individual patient meta-analyses suggest that co-occurring emotional problems do not attenuate the impact of group parenting programmes for DBDs (Leijten et al. 2020). However, whilst there is excellent evidence demonstrating the efficacy of behavioral parenting interventions for DBDs (Fonagy et al. 2015), there is little guidance to suggest the optimal treatment for children with DBDs and co-occurring internalizing disorders. Previous evidence suggests that treating the co-occurring anxiety disorder with cognitive behavioral therapy for anxiety is also effective in reducing symptoms of DBDs (Kreuze et al. 2018; Mahdi et al. 2019). However, it is not known whether treatment for behavioral disorders impacts on co-occurring internalizing disorders.

Given the high comorbidity between conduct and internalizing disorders, it is clinically relevant to explore the optimal treatment strategy (Loeber et al. 2000; Stalk et al. 2015). Therapists may prioritise treatment of externalizing over internalizing problems when faced with comorbidity (Milette-Winfree & Mueller, 2018) but it is not clear whether research evidence supports such an approach. As internalizing disorder interventions are effective in reducing co-occurring externalizing behavior problems (Mahdi et al. 2019), it may be that the opposite is also true. The aim of the review was therefore to determine the impact of behavior interventions for the treatment of disruptive behavior in children and young people on comorbid internalizing symptoms.

## Method

A systematic literature review was performed according to the Cochrane guidelines for systematic reviews (Higgins, Thomas, Chandler, Cumpston, Li, Page et al. 2019). The review was registered on the PROSPERO International Register of Systematic reviews (CRD42020176693).

The databases PsycINFO, EMBASE and MEDLINE were searched in January 2021 using the following search terms: ((triple p or incredible years or parenting program*) and (adolescen* or teen* or child* or toddler*) and (conduct disorder or oppositional defiant disorder or child behavi* or agress* behavi* or antisocial behavi*) and (double-blind or random* assigned or control)). There were no date restrictions on records retrieved. The records retrieved from this search were screened for eligibility based on the inclusion and exclusion criteria described below. Reference lists and citations of included articles were also searched for relevant articles.

### Inclusion Criteria

#### Participants

i. Children and adolescents between the ages of 0 and 18
ii. Participants who were either given a DBD diagnosis according to the Diagnostic and Statistical Manual (DSM) 3^rd^ revised edition, 4^th^ and 5^th^ edition (American Psychiatric Association, 1987, 2000, 2013) or the International Statistical Classification of Diseases, 10th revision (ICD 10; World Health Organization, 2004) or scored at clinical level on a standardized validated measure of disruptive behavior symptoms, such as the Strengths and Difficulties Questionnaire (SDQ; Goodman, 1999), the Eyberg Child Behavior Inventory (ECBI; Eyberg & Pincus, 1999) or the Achenbach Child Behavior Checklist (CBCL; Achenbach & Rescorla, 2000). Participants were not required to have internalizing symptoms above a clinical threshold.

##### Intervention

i. Parent training interventions targeting elevated externalizing behavior problems or DBDs

##### Comparator

i. Randomized controlled trials with a treatment as usual or waitlist control

##### Outcome

i. A measure of externalizing behavior problems
ii. A measure of internalizing symptoms

### Exclusion Criteria

i. Studies assessing non-behavioral interventions, such as music therapy
ii. Studies that focused on the treatment of conditions other than DBDs, such as ADHD, or anxiety disorders
iii. Studies not available in English
iv. Trials on young people with no or sub-threshold disruptive behavior concerns

### Study Selection

Study selection was performed by comparing all records against the aforementioned inclusion and exclusion criteria. This process was independently performed by two reviewers (EZ & DP). Qualified clinical psychologists (RS & SB) were consulted to resolve any disagreements and discrepancies concerning the final selection of eligible studies.

### Data Extraction

A data extraction form was developed, consisting of the study and sample characteristics, and the main outcomes. Data was extracted independently by two reviewers (EZ & DP). Interrater reliability was 80%.

### Assessment of Risk of Bias

An assessment of risk of bias was performed independently by two reviewers (EZ & DP) using The Effective Public Health Practice Project (EPHPP, 2019a, b). Cohen’s kappa for interrater reliability was 75% (McHugh, 2012).

Each study was initially evaluated as strong, moderate or weak on the following domains: selection bias, study design, confounders, blinding, data collection methods, withdrawals and dropouts, based on EPHPP criteria. A final rating score was then assigned to each study based on the total number of domains that were scored as weak. Studies with no weak ratings were considered strong; those with one weak rating were deemed moderate, while those with two or more weak ratings were regarded as weak (Higgins et al. 2011).

## Results

**Fig. 1:**
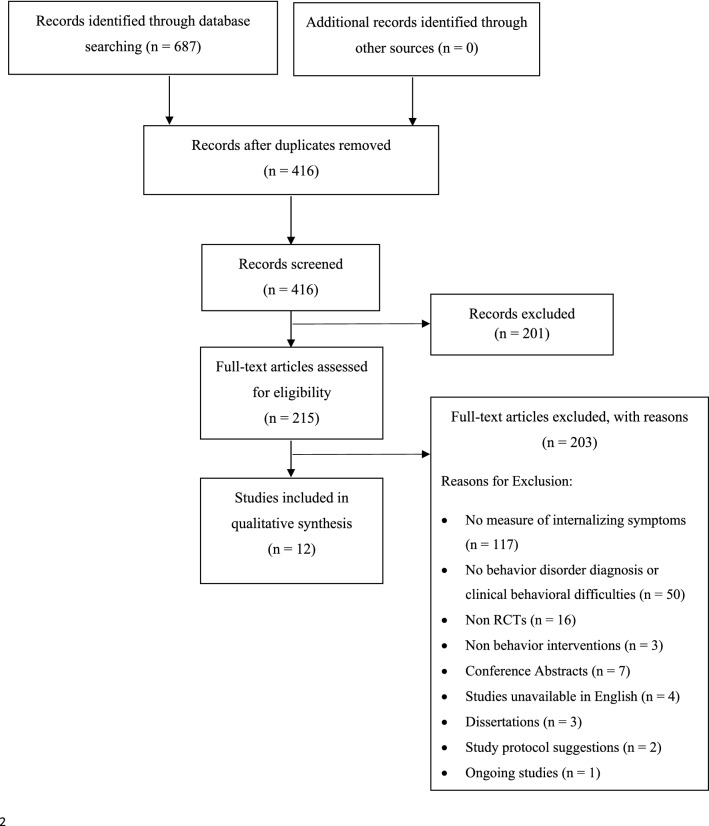
Prisma diagram

The initial search identified 687 records, of which 416 remained after duplicates were removed. A total of 12 met the criteria for inclusion in the current systematic review. A flow chart of the search process stages along with reasons for inclusion and exclusion can be found in Fig. 1. Details on the selected studies’ characteristics can be found in Table 1.

**Table 1:** Study and sample characteristics

Study	Primary disorder	Intervention	Interventionist	Study type	Sample size (#male)	Age (M, SD)	Study location	Quality rating
Axberg and Broberg (2012)	ODD (DSM diagnosis)	IY Basic Parent Training	Certified IY BASIC Trainer	RCT	62 (52)	4–8 yrs (N/A)	Sweden	Moderate
Baker et al (2017)	SDQ scores = / > 14	TP Online Brief	Online	RCT	200 (110)	2–9 yrs (4.4, 1.9)	Australia	Moderate
Larsson et al (2009)	ODD, CD (DSM diagnosis)	IY Parent Training & IY Child Training	IY-Trained Therapists	RCT	127 (101)	4–8 yrs (6.4, 1.5)	Norway	Strong
Meybodi et al (2019)	CBCL externalizing subscale score = / > 65	TIK	Doctoral tutor facilitators	RCT	54 (32)	3–6 yrs (4.33, 0.93)	Iran	Moderate
Morpeth et al (2017)	SDQ scores = / > 17	IY Basic Parent Training	Trained IY facilitators	RCT	161 (101)	3–5 yrs (3.7, 6)	UK	Strong
Palmer et al (2019)	ECBI Intensity score = / > 45	TP	First author trained on triple P delivery	RCT	78(50)	5–8 yrs (N/A)	New Zealand	Weak
Patterson et al (2002)	ECBI Intensity score = / > 100	IY Basic Parent Training	Certified IY BASIC Trainer	RCT	116 (N/A)	2–8 yrs (N/A)	Oxford, UK	Strong
Sanders et al (2012)	ECBI Intensity score = / > 131 ECBI Problem score = / > 15	TP Online	Online	RCT	116(78)	2–9 yrs (4.7, 1.7)	Australia	Weak
Schappin et al (2013)	CBCL externalizing subscale score = / > 60	TP	Social workers, registered health care psychologists and registered clinical psychologists trained on TP delivery	RCT	67(40)	2–5 yrs (2, 1)	Netherlands	Moderate
Scott et al (2010)	SDQ conduct problem score = / > 5 DSM 4 Score = / > 10 for ODD	IY Basic Parent Training & Child Literacy Programme	IY: Psychology graduates with an IY group leader certification, senior mental health nurses with an IY mentor certification, senior family therapists For CLP: Teacher with experience in remedial reading	RCT	112 (79)	5–6 yrs (5.21, 0.31)	London, UK	Strong
Webster-Stratton and Herman (2008)	ODD or CD (DSM 3-R or 4 diagnosis) ECBI score > than 2SD above the normal cutoff	IY Basic Parent Training	Mental health professionals trained on IY delivery	RCT	181(142)	3–8.5 yrs (5.25, 1.2)	USA	Moderate
Wiggins et al (2009)	Abnormal scores on at least one SDQ problem subscale	TP	TP-trained therapists	RCT	60 (46)	4–10 yrs (6.2, 2.2)	Australia	Weak

*M* mean, *SD* standard deviation, *Yrs* years, *N/A* not assigned, *IY* incredible years, *TP* triple P, *TIK* tuning in to kids, *RCT* randomized control trial, *SDQ* Strengths and Difficulties Questionnaire, *ECBI* Eyberg Child Behavior Inventory, *CD* conduct disorder, *ODD* Oppositional Defiant Disorder, *DSM* Diagnostic and Statistical Manual for Mental Disorders, *CBCL* Achenbach Child Behavior Checklist

### Quality Assessment

According to the EPHPP, four of the twelve studies were deemed strong (Larsson et al. 2009; Morpeth et al. 2017; Patterson et al. 2002; Scott et al. 2010); another five were rated as moderate (Axberg & Broberg, 2012; Baker et al. 2017; Meybodi et al. 2019; Schappin et al. 2013; Webster-Stratton & Herman, 2008), and the remaining three were deemed weak (Palmer et al. 2019; Sanders et al. 2012; Wiggins et al. 2009) (Table 1). Cohen’s kappa for interrater reliability was high at 75% (McHugh, 2012).

### Study Characteristics

#### Participants

A total of 1334 children and young people were enrolled in the studies. In all studies reviewed, the majority of participants were male, except for one study in which the sample’s gender distribution is not mentioned (Patterson et al. 2002). Participants’ ages ranged between 2 and 10 years, with a mean age of 5 years. In only one study did participants receive a formal, secondary internalizing disorder diagnosis (Webster-Stratton & Herman, 2008). In the remaining 11 studies, 3 had samples with a mean internalizing score above the clinical threshold for an internalizing disorder at baseline (Meybodi et al. 2019; Morpeth et al. 2017; Schappin et al. 2013).

#### Intervention

The majority of the studies in this review tested the efficacy of the Incredible Years (Axberg & Broberg, 2012; Larsson et al. 2009; Morpeth et al. 2017; Patterson et al. 2002; Scott et al. 2010; Webster-Stratton & Herman, 2008) and the Triple P (Baker et al. 2017; Palmer et al. 2019; Sanders et al. 2012; Schappin et al. 2013; Wiggins et al. 2009) parent training intervention programs. Scott and colleagues (2010) assessed the Incredible Years parent training program combined with the Child Literacy Program. Finally, one study assessed the Tuning In To Kids parent training intervention (Meybodi et al. 2019).

The majority of the interventions were delivered face to face and were led by professionals qualified to deliver each intervention (Axberg & Broberg, 2012; Larsson et al. 2009; Meybodi et al. 2019; Morpeth et al. 2017; Palmer et al. 2019; Patterson et al. 2002; Schappin et al. 2013; Scott et al. 2010; Webster-Stratton & Herman, 2008; Wiggins et al. 2009). In two studies on the Triple P intervention, the program was delivered online (Baker et al. 2017; Sanders et al. 2012). The protocols for each intervention used in the studies were similar in format, number of sessions and overall duration as all intervention procedures were conducted in accordance with the relevant manualized guidelines indicated for each intervention (The Incredible Years, 2013; Triple P International, 2020; Tuning In To Kids, 2020). On average, the interventions lasted 10–12 sessions and the majority of those, except for those assessing online interventions, were conducted in group format (Axberg & Broberg, 2012; Larsson et al. 2009; Meybodi et al. 2019; Morpeth et al. 2017; Palmer et al. 2019; Patterson et al. 2002; Schappin et al. 2013; Scott et al. 2010; Webster-Stratton & Herman, 2008; Wiggins et al. 2009).

#### Comparators

In the majority of studies (10/12) the intervention groups were compared to a waitlist control group (Axberg & Broberg, 2012; Baker et al. 2017; Larsson et al. 2009; Meybodi et al. 2019; Morpeth et al. 2017; Patterson et al. 2002; Schappin et al. 2013; Scott et al. 2010; Webster-Stratton & Herman, 2008; Wiggins et al. 2009). One study assessing the Triple P Online Intervention included an ‘internet use as usual’ control group (Sanders et al. 2012). One study compared a sufficient exemplar Triple P discussion group (providing examples of how to apply parenting strategies to promote generalization of skills learnt to different situations) to a single exemplar, narrowly focused control group – the Triple P Dealing with Disobedience Discussion Group (Palmer et al. 2019).

#### Outcome

The primary DBD diagnosis was established according to the DSM criteria (Axberg & Broberg, 2012; Larsson et al. 2009; Scott et al. 2010; Webster-Stratton & Herman, 2008) or based on clinically elevated scores on validated disruptive behavior measures such as the ECBI (Palmer et al. 2019; Patterson et al. 2002; Sanders et al. 2012; Webster-Stratton & Herman, 2008), the SDQ (Baker et al. 2017; Morpeth et al. 2017; Scott et al. 2010; Wiggins et al. 2009) and the CBCL (Meybodi et al. 2019; Schappin et al. 2013) scales.

To measure externalizing symptoms from pre- to post-treatment and at follow up, the majority of studies used the ECBI (Axberg & Broberg, 2012, Baker et al. 2012, Larsson et al. 2009; Meybodi et al. 2019; Morpeth et al. 2017; Palmer et al. 2019; Patterson et al. 2002; Sanders et al. 2012; Schappin et al. 2013; Scott et al. 2010; Webster-Stratton & Herman, 2008). Other measures used were the SDQ (Morpeth et al. 2017; Patterson et al. 2002; Sanders et al. 2012) and the CBCL (Schappin et al. 2013; Wiggins et al. 2009). Two studies used direct observations of child behavior and parent–child interactions (Sanders et al. 2012; Scott et al. 2010); one used teacher reports of child behavior (Schappin et al. 2013); one (Baker et al, 2017) used the Child Adjustment and Parent Efficacy Scale (CAPES) (Morawska et al. 2014) with the remaining study using the Parent Account of Child Symptoms (PACS) scale (Scott et al. 2010).

Internalizing scores were, in most studies, measured via the relevant internalizing symptom subscales on the SDQ (Axberg & Broberg, 2012; Morpeth et al. 2017; Palmer et al. 2019; Patterson et al. 2002; Sanders et al. 2012) and the CBCL (Larsson et al. 2009; Schappin et al. 2013; Webster-Stratton & Herman, 2008; Wiggins et al. 2009). Other measures included the CAPES (Baker et al. 2017), the ECBI (Schappin et al. 2013), the PACS (Scott et al. 2010) and the Persian version of the Emotion Regulation Checklist (ERC) (Meybodi et al. 2019).

### Intervention Efficacy on Primary Externalizing and Comorbid Internalizing Symptoms

#### The Incredible Years Program

##### Primary Externalizing Behavior Problems

All six studies assessing the efficacy of the Incredible Years Program indicated significant reductions on ECBI behavior problem and intensity scores from pre- to post-treatment and at subsequent follow ups. These reductions were deemed clinically and statistically significant both within the parent training intervention group and between the intervention and control groups. Incredible Years effectively reduced both the number and the frequency of disruptive behavior problems (Axberg & Broberg, 2012; Larsson et al. 2009; Morpeth et al. 2017; Patterson et al. 2002; Scott et al. 2010; Webster-Stratton & Herman, 2008).

##### Comorbid Internalizing Symptoms

Four of the six studies assessing the Incredible Years found significant reductions in internalizing scores (Larsson et al. 2009; Morpeth et al, 2017; Patterson et al, 2002; Webster-Stratton & Herman, 2008). Only one of these included participants with a formal, secondary internalizing disorder diagnosis at baseline (Webster-Stratton & Herman, 2008). One study found significant reductions in mother but not father reported CBCL internalizing scores for the intervention group from pre- to post-treatment and follow-up compared to the control group (Larsson et al. 2009). Patterson and colleagues (2002) found that although SDQ internalizing scores were reduced from pre- to post-treatment for the intervention group, these effects were not maintained at the subsequent 6-month follow up, where scores for the control group were lower. Two studies indicated no significant reduction in internalizing symptom scores from pre- to post-treatment and follow up, neither within the Incredible Years intervention group nor between the intervention and control groups (Axberg & Broberg, 2012; Scott et al. 2010).

#### The Triple P Program

##### Primary Externalizing Behavior Problems

Five studies assessed the efficacy of the Triple P program (Baker et al. 2017; Palmer et al. 2019; Sanders et al. 2012; Schappin et al. 2013; Wiggins et al. 2009), with three of these considered ‘weak’ according to the quality rating (Palmer et al. 2019; Sanders et al. 2012; Wiggins et al. 2009). Of these five, three indicated that Triple P led to significant reductions on the ECBI behavior problem and intensity scale scores, both between the Triple P condition and the control group and within the Triple P group from pre- to post-treatment and follow up (Baker et al. 2017; Palmer et al. 2019; Sanders et al. 2012). However, in Palmer and colleagues’ (2019) study, these intervention effects were significant only in mother, not father reports of child conduct problems. One study indicated significant reductions on the CBCL externalizing problem scores (Wiggins et al. 2009), while Sanders and colleagues (2012), also found significant reductions on both the SDQ externalizing problem scores and observed child conduct problems between the intervention condition and control group from pre- to post-treatment and follow up. Finally, one study found no significant differences in child behavioral outcomes between the Triple P intervention group and the control group nor within the Triple P group across the different time points. In fact, behavior problems decreased slightly, non-significantly in both groups (Schappin et al. 2013).

##### Comorbid Internalizing Symptoms

Of the five studies looking at the efficacy of Triple P, only three found significant reductions in comorbid internalizing scores at post treatment and follow up compared to baseline and all three of these were considered ‘weak’ studies according to the quality ratings (Palmer et al. 2019; Sanders et al. 2012; Wiggins et al. 2009). Of these three, two studies reported significantly lower scores on the SDQ emotional subscale from pre- to post-treatment and follow up for the Triple P intervention condition, but not for the control condition (Palmer et al. 2019; Sanders et al. 2012). Wiggins and colleagues (2009) indicated significantly lower internalizing symptom scores on the CBCL for the intervention condition. However, the difference in CBCL scores between the control and intervention conditions were not statistically significant (Wiggins et al. 2009).

Finally, two studies indicated no significant reductions in co-occurring internalizing symptom scores from pre- to post-treatment and follow up, neither within the Triple P group nor between the control and intervention groups (Baker et al. 2017; Schappin et al. 2013).

#### The Tuning In To Kids Program

##### Primary Externalizing Behavior Problems

One study assessed the efficacy of the Tuning In To Kids program (Meybodi et al. 2019). Results indicated significant reductions in mother reported child behavior problems on the ECBI problem and intensity scores from pre- to post-treatment and follow up in the Tuning In To Kids intervention condition only. Results were also significant between the intervention and control group as mothers in the control condition did not report significant reductions in child conduct problems (Meybodi et al. 2019).

##### Comorbid Internalizing Symptoms

In Meybodi et al.’s (2019) study assessing Tuning In To Kids, scores on the ERC significantly improved and decreased from pre- to post-treatment in the intervention group compared to the control group.

## Discussion

### Main Findings

The purpose of the current review was to determine the impact of parent training intervention programs specifically aimed at treating externalizing behavior problems on comorbid internalizing symptoms in children and adolescents.

A total of twelve Randomized Controlled Trials were reviewed. Overall, the results indicated that parent training programs designed to treat conduct problems may positively affect co-occurring internalizing concerns; Seven of the twelve studies found significant reductions in internalizing symptoms in the intervention group compared to the control group, from pre- to post-treatment and at subsequent follow ups. However, only one (Webster-Stratton & Herman, 2008 – rated ‘moderate’ on quality) included children with a diagnosis of a cooccurring anxiety disorder. Three of the seven studies finding significant reductions were considered weak. Of the seven demonstrating significant reductions in internalizing symptoms, three used the Incredible Years program (Larsson et al. 2009; Morpeth et al. 2017; Webster-Stratton & Herman, 2008), one used the Tuning In To Kids programme (Meybodi et al, 2019) and the remaining three used the Triple P program (Palmer et al. 2019; Sanders et al. 2012; Wiggins et al. 2009). One study on the Incredible Years program indicated reductions in comorbid internalizing symptom measures at post treatment, however these effects were not maintained at follow up (Patterson et al. 2002). The remaining four studies demonstrated some reductions in internalizing symptoms in the intervention versus the control conditions, but these reductions were not statistically significant despite significant reductions in externalizing symptoms compared to the control group. Two of these studies assessed the Incredible Years intervention (Axberg & Broberg, 2012; Scott et al. 2010) and another two the Triple P intervention (Baker et al. 2017; Schappin et al. 2013). All four studies received moderate or strong quality ratings, supporting the reliability and validity of their results: Three were deemed moderate (Axberg & Broberg, 2012; Baker et al. 2017; Schappin et al. 2013) while the remaining one was deemed strong (Scott et al. 2010) according to the quality assessment. However, in none of these four studies did the subjects meet the diagnostic criteria to receive a secondary internalizing disorder diagnosis nor were their internalizing symptoms significantly elevated at baseline. Therefore, the non-significant reductions in internalizing symptom measures found in these four studies may be due to symptoms being low from baseline, making it harder to identify change.

### Mechanisms of Change

The results of this systematic review suggest that interventions focused on treating primary behavior disorder diagnoses or clinical-level behavioral symptoms may also have a positive impact on comorbid internalizing symptoms. A number of potential mechanisms may explain this effect. DBDs often present with internalizing symptoms (Burcusa et al. 2003; Angold, Costello & Erkanli, 2003) and the underlying psychopathology of the two conditions may overlap. For example, evidence has indicated that negative emotionality may be a risk factor for both categories of disorders (Nigg & Huang-Pollock, 2003; Wolff & Ollendick, 2006). Therefore, certain basic aspects of behavior interventions may be applied to benefit the treatment of various conditions. Empirical evidence by Brumariu and Kerns (2010) suggested a link between the quality of the parent–child attachment and the development of internalizing symptoms. In particular, insecure attachment styles (avoidant, anxious or disorganized) were linked to a greater likelihood of developing anxiety and mood disorders like depression (Brumariu & Kerns, 2010).

Internalizing symptoms may also present as a consequence of externalizing behavior problems (Fonagy et al. 2015). Research by Gilliom and Shaw (2004) found that early childhood externalizing problems were followed by later internalizing symptom development. Intervention for DBDs may eliminate the secondary, comorbid internalizing symptoms that stem from the primary disorder. However, ten of the twelve studies had a waitlist control and not an active control. Further research with active control groups would enhance understanding of the mechanisms of change.

### Strengths of this Systematic Review

The present systematic literature review is the first to address the efficacy of behavior-focused parent training interventions on comorbid conditions. The findings of this review partially confirm the initial hypothesis that parent training treatment strategies that are aimed at targeting externalizing behavior problems may also successfully improve comorbid internalizing symptoms. Given the high prevalence of behavioral problems in children and adolescents (Hood & Eyberg, 2003; Katzmann et al. 2019), in addition to the high comorbidity between internalizing and externalizing behavior problems in young people (Fonagy et al. 2015), the data from this systematic review effectively addresses and responds to a gap in the scientific literature regarding appropriate treatment strategies to manage such comorbidities.

Among the strengths of the present review is the replicable search strategy applied based on the Cochrane guidelines for systematic reviews (Higgins et al. 2019). Extensive and specific search terms were used to cover all pertinent ages, interventions, primary diagnoses and comorbidities, in order to maximize the relevance of the articles retrieved. Strict inclusion and exclusion criteria were applied to reliably determine eligibility. Records were screened for eligibility by two independent reviewers to support the validity and reliability of the final set of articles included in the review. The review includes RCTs only.

### Limitations and Suggestions for Future Research

In addition to the strengths and limitations of the included studies described above, the review itself also had some limitations. Firstly, in almost all of the records assessed, comorbid internalizing symptoms were secondary outcomes and were usually not mentioned in the abstracts or key terms of the studies. It is therefore possible that some studies were missed in the original search.

Secondly, the current review assessed intervention efficacy in reducing co-occurring internalizing symptom measures. However, participants had a formal secondary internalizing disorder diagnosis in only one study (Webster-Stratton & Herman, 2008). In the majority of studies, internalizing symptom measures were assessed through reports such as the SDQ, ECBI or CBCL. These internalizing symptom measures are not specific to particular disorders and it may be useful for future research to use more distinct measures of internalizing problems in future trials of disruptive behaviour interventions. In addition, in most studies, scores on these measures were below clinical cutoffs at baseline. This may limit both the power to find a true effect if one exists and the ability to conclude whether interventions targeting behavior disorders are also able to target co-occurring internalizing disorders as opposed to symptoms alone. Future research should also consider evidence from studies in which subjects score above clinical cutoffs or have a formal diagnosis for both externalizing and internalizing conditions.

### Clinical Implications

The current systematic literature review indicated the efficacy of the Incredible Years, Triple P and Tuning In To Kids parent training behavior interventions on untargeted comorbid internalizing symptoms in young people. Given the high rates of comorbidity between externalizing and internalizing conditions in children and adolescents, knowledge on appropriate intervention strategies to treat both primary and secondary diagnoses is important to be applied in clinical practice (Stalk et al. 2015). The majority of research on psychiatric comorbidity so far has focused on using transdiagnostic treatment approaches targeting both conditions simultaneously or applying distinct interventions to target each one of the comorbid conditions separately (Marchette & Weisz, 2017). However, evidence from this review suggests that disorder-specific interventions may also be effective in targeting cooccurring symptoms. A disorder-specific intervention approach may allow for increased treatment adherence and fidelity both due to their time and cost-effectiveness and because following multiple treatment protocols at once may be overwhelming and has been linked to higher dropout rates (Dretzke, Frew, Davenport, Barlow, Stewart-Brown, Sandercock et al. 2005; Dretzke et al. 2009; Levy et al. 2007). It may be helpful to see whether internalizing symptoms improve in response to treatment of DBD treatment and then consider whether additional treatment for internalizing problems is needed, and vice versa. Therefore, clinicians should be informed on the benefits of disorder-specific interventions in order to apply them in practice.

## Conclusion

The findings of the current systematic review overall confirm the robust efficacy of the Incredible Years, Triple P and Tuning In To Kids parent training behavior interventions in reducing externalizing behavior difficulties in children and adolescents presenting with DBDs or elevated behavior problem scores that are within the clinical range. Although findings for comorbid internalizing symptoms were less consistent, internalizing symptom measures were significantly reduced in the majority of the studies assessed. Therefore, parent training programs targeted at the treatment of externalizing behavior problems may also effectively address comorbid internalizing symptoms at the same time.

## Data Availability

Data are available upon reasonable request.
